# Intracellular pH Regulates Cancer and Stem Cell Behaviors: A Protein Dynamics Perspective

**DOI:** 10.3389/fonc.2020.01401

**Published:** 2020-08-26

**Authors:** Yi Liu, Katharine A. White, Diane L. Barber

**Affiliations:** Department of Cell and Tissue Biology, University of California, San Francisco, San Francisco, CA, United States

**Keywords:** intracellular pH, cancer, stem cell, protein structure, protonation

## Abstract

The International Society of Cancer Metabolism (ISCaM) meeting on Cancer Metabolic Rewiring, held in Braga Portugal in October 2019, provided an outstanding forum for investigators to present current findings and views, and discuss ideas and future directions on fundamental biology as well as clinical translations. The first session on *Cancer pH Dynamics* was preceded by the opening keynote presentation from our group entitled *Intracellular pH Regulation of Protein Dynamics: From Cancer to Stem Cell Behaviors*. In this review we introduce a brief background on intracellular pH (pHi) dynamics, including how it is regulated as well as functional consequences, summarize key findings included in our presentation, and conclude with perspectives on how understanding the role of pHi dynamics in stem cells can be relevant for understanding how pHi dynamics enables cancer progression.

## Introduction

Intracellular pH (pHi) was previously thought to be mostly constant for cellular homeostasis and possibly dysregulated in diseases. We now know, however, that pHi is dynamic in normal cells and clearly dysregulated in a number of diseases. In normal cells, pHi changes during cell cycle progression, increasing ~0.3–0.4 pH units at the end of S phase and if this increase is blocked, G2/M is delayed with increased inhibitory phosphorylation of Cdk1-Tyr15 and suppressed cyclin B1 expression (1–3). Additionally, pHi dynamics regulates cell-substrate adhesion remodeling and migration, with increased pHi enabling both behaviors (4–7). Emerging evidence also indicates a critical role for increased pHi in epithelial plasticity, including epithelial to mesenchymal transition (EMT) (8), and stem cell differentiation (9–12). Moreover, it is now well-established that dysregulated pHi is seen with many diseases, most notably cancers, which often have a constitutively increased pHi (13–18), and neurodegenerative disorders, which are associated with a constitutively decreased pHi (19, 20). Our review focuses on dysregulated pHi dynamics in cancer; however, another feature of cancers is a dysregulated extracellular pH that is lower (~7.0) compared with normal tissues (~7.4).

Although many factors contribute to pHi dynamics, the major regulators in most mammalian cells are plasma membrane ion exchangers, including the Na^+^-H^+^ exchanger NHE1, the Na^+^-${\text{HCO}}_{3}^{-}$ transporter NBC, and the Na^+^-dependent Cl^−^-${\text{HCO}}_{3}^{-}$ transporter NDCBE, which are acid-extruders, and Cl^−^-${\text{HCO}}_{3}^{-}$ exchangers of the anion exchanger (AE) family, which are acid loaders (21–23). The BioParadigms Solute Carrier tables^1^ are an excellent resource on the classification, expression, and transport characteristics of these ion exchangers. Additional plasma membrane ion transport proteins that contribute to pHi dynamics, albeit to less of an extent, include V-ATPases and monocarboxylate transporters of the MCT family. The broad range of ion transport proteins regulate pHi dynamics through changes in their expression and activity, the latter mostly mediated by posttranslational modifications as many are substrates of key signaling kinases, including for NHE1, p90rsk (24), Akt (25, 26), the Rho kinase ROCK (27), and the Ste20 kinase MAP4K4 (28), previously termed NIK. Experimentally, these exchangers can be pharmacologically or genetically targeted to understand how they contribute to pHi dynamics and how pHi dynamics regulates cell behaviors.

We have a relatively strong understanding of how changes in pHi are generated and the effects of pHi changes on myriad cell functions. However, a mechanistic understanding of how pHi changes regulate cell behaviors remains understudied, particularly effects on signaling networks and protein functions. At the ISCaM meeting we presented our work on how changes in pHi regulate protein dynamics to enable cancer and stem cell behaviors, which we summarize in this review. Key to pH-regulated protein structure and function is considering protonation and deprotonation as a protein posttranslational modification, analogous to posttranslational modification by phosphorylation, acetylation, and methylation as we previously described (29). However, studying protonation and deprotonation as a posttranslational modification is more difficult compared with other posttranslational modifications because it is not catalyzed by an enzyme and cannot be detected by mass spectrometry or antibodies. Furthermore, many endogenous “pH sensors” or proteins that are regulated by pH dynamics within the cellular range are coincidence (AND-gate) detectors with their structural conformations, activities, or binding affinities dependent on multiple posttranslational modifications, most commonly phosphorylation or dephosphorylation and protonation or deprotonation.

## Intracellular pH and Cancer Cell Behaviors: From the Protein View

**Figure 1:**
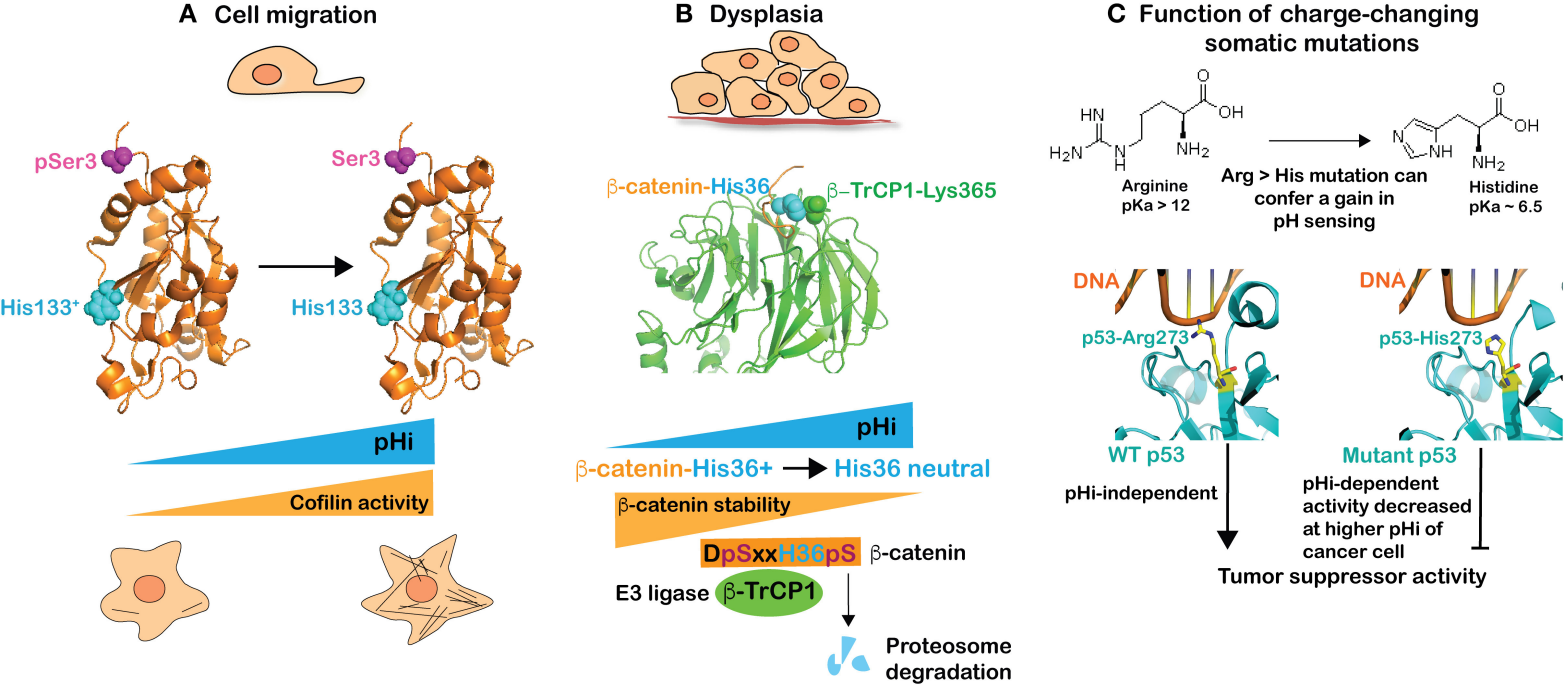
The higher pHi of cancer cells enables many behaviors, including directional migration and tumorigenesis as well as the tumorigenic functions of proteins with charge-changing arginine to histidine mutations. **(A)** Cell migration is in part dependent on increased activity of cofilin with increased pHi. Cofilin is a coincidence-regulated pH sensor that is activated by deprotonation of His133 (cyan) and dephosphorylation of Ser3 (magenta) for actin polymerization enabling cell migration. **(B)** Dysplasia is associated with increased pHi, which decreases β-catenin stability. β-catenin is a coincidence-regulated pH sensor with deprotonation of His36 (cyan) and phosphorylation of Ser33/37 by GSK3β enabling binding to the E3 ligase β-TrCP1 for targeting to the proteasome for degradation. Crystal structure data show that β-catenin-His36 is in close proximity to β-TrCP1-Lys365, which suggest that binding would be electrostatically unfavorable with a protonated His36 at lower pHi. **(C)** Charge changing somatic mutations can confer pH-regulated protein activity. Structure of wild-type p53 (left) and mutant p53-R273H (right) in complex with DNA indicating an electrostatic interaction of Arg273 with the negatively charged phosphate-backbone of DNA that could be partially enabled by protonated, but not neutral, His273.

Most cancer cells have a higher pHi compared with untransformed cells, regardless of the mutational landscape or tissue origin. This higher pHi enables many cancer behaviors, including increased proliferation, directional migration, tumorigenesis, and most recently recognized, the oncogenic and tumor-suppressor functions of proteins with charge-changing mutations (Figure 1). At the ISCaM meeting we presented our findings on pH sensors regulating cell migration and tumorigenesis as well as how pHi dynamics in cancer cells affect the functions of proteins with somatic mutations encoding arginine to histidine substitutions.

*Cell migration* is confirmed to be regulated by pHi in many cell types and species (6, 30–34). An increased pHi of ~0.3–0.4 units is seen in migrating cells and preventing the increased pHi inhibits migratory rate and directionality, and impairs cell polarity. Our presentation described several pH sensors we identified in atomistic detail that collectively regulate different aspects of migration. These include guanine nucleotide exchange factors for the low molecular weight GTPase Cdc42 involved in cell polarity (35), talin binding to actin filaments (36), and focal adhesion kinase (FAK) activity for cell-substrate adhesion dynamics (5) as well as cofilin for actin polymerization (37). The single histidine in cofilin, His133 (human), has an upshifted pKa to ~ 7.2 and must be neutral for increased cofilin activity (Figure 1A). However, cofilin is a coincidence detector and full activity also requires dephosphorylation of Ser3 (Figure 1A) by one of several phosphatases, which releases an autoinhibited interaction between phosphorylated serine and lysine 126 and 127 to allow binding to actin filaments. This AND-gate regulation enables signaling mechanisms to increase cofilin activity in time (with migratory cues) and space (at the leading edge of a migrating cell), and highlights that for many pH sensors a change in protonation state does not function as a binary switch.

*Tumorigenesis and dysplasia* are enabled by increased pHi regulated by NHE1, NBCs, and MCTs, including tumor cell proliferation, growth, and survival (38–40). Our presentation included two of our recent key findings on pHi and tumorigenesis. First, that increased pHi from ~7.30 to ~7.65 in Drosophila eye epithelia by overexpressing Drosophila *dnhe2*, an ortholog of mammalian NHE1, is sufficient to induce dysplasia in the absence of an activated oncogene (41). Second, that β-catenin, an adherens junction and Wnt pathway protein, is a pH sensor, with pHi not regulating its activity but rather its stability, which decreases at pHi > 7.5 (42). Using a phenotype screen, we found that overexpressing β-catenin suppresses dysplasia in Drosophila eye epithelia with constitutively increased pHi induced by overexpression of *dnhe2*. These data suggested a lower abundance of β-catenin at higher pHi, which we confirmed in mammalian cells. We also resolved the pH sensing mechanism of His36 (human) in the N-terminus of β-catenin, which when neutral (at higher pHi) increases binding affinity for the E3 ligase β-TrCP1. However, like cofilin described above, β-catenin is a coincidence detector requiring both a neutral His36 and phosphorylated flanking Ser33 and Ser37 for binding β-TrCP1 (Figure 1B). The role of phosphorylated serines in enabling proteasome-mediated degradation of β-catenin has long been recognized (43). The importance of a neutral His36 for binding β-TrCP1 is evident in the crystal structure of β-TrCP1in complex with an N-terminal β-catenin peptide (44) (PDB: 1P22), which shows the proximity of β-catenin-His36 and β-TrCP1-Lys365 (Figure 1B). This suggests that binding would be electrostatically unfavorable with a protonated His36 at lower pHi. Importantly, the DSxxHS motif is conserved in β-catenin across species and occurs in a number of other β-TrCP1 target proteins (45), including the transmembrane protein polycystin 2, the tumor suppressor tensin 2, the centrosomal protein Cep97, the hedgehog pathway protein Gli3, and myosin-XVIIIa, suggesting these substrates may have similar pH sensitive binding to β-TrCP1 and regulated protein stability. We also described that a cancer-associated somatic mutation, β-catenin-H36R, is insensitive to pHi-regulated degradation and, when expressed in Drosophila eye epithelia, enhances Wnt pathway activity, causes tissue overgrowth growth, and induces ectopic tumors. With this mutation, β-catenin stability could be retained at the higher pHi of a cancer cell and enable tumorigenesis. As described in the section below, this is an example of a charge-changing mutation that confers a loss of pH sensing.

*Charge-changing somatic mutations* can confer a change in pH sensing and enable cancer behaviors specifically at increased pHi. We recently showed that recurrent arginine to histidine mutations in p53 and EGFR can confer a gain in pH sensing to the mutant proteins. Arginine, with a pKa of 12, will be protonated regardless of pHi while histidine, with a pKa near neutral, can titrate with cellular changes in pHi. We found that a highly recurrent arginine to histidine mutation in the tumor suppressor p53 (p53-R273H) could confer pH-dependent DNA binding and transcription of p53 target genes, with decreased transcription at a higher pHi of 7.6 compared with 7.2 (46). The crystal structure of wild-type p53 (47) (PDB: 4HJE) and mutant p53-R273H (48) (PDB: 4IBW) in complex with DNA suggests that wild-type Arg273 forms an electrostatic interaction with the negatively charged phosphate-backbone of DNA (Figure 1C). At the lower pHi of a non-transformed cell, His273 is likely protonated and retains some binding to the negatively-charged DNA but, at the higher pHi of a cancer cell, His273 is likely deprotonated, reducing DNA binding and expression of p53 target genes (Figure 1C). Importantly, lowering pHi in cancer cells expressing p53-R273H recovered p53 transcriptional activity and p53-dependent cell death in response to double-strand breaks (46). We also showed that a cancer-associated arginine to histidine substitution in the epidermal growth factor receptor (EGFR-R776H) that is recurrent in lung cancers confers pH sensing to the mutant protein. Increasing pHi from 7.2 to 7.6 increases activity of EGFR-R776H but not wild-type receptor, and increases cell proliferation and cellular transformation in cells expressing the mutant but not wild-type receptor (46). These results suggest that charge-changing mutations can confer a gain in pH-sensing not seen with the wild-type protein. This work also indicates that charge-changing somatic mutations can confer dynamic function to mutant proteins, specifically inactivating a tumor suppressor and specifically activating an oncogene at the increased pHi of cancer.

## Intracellular pH and Epithelial Plasticity: Focus on Stem Cell Differentiation

Recent findings indicate that pHi dynamics is a key regulator of epithelial plasticity, with increased pHi enabling EMT (8) and epithelial branching morphogenesis (49) as well as differentiation of melanocytes (50), embryonic and adult stem cells (9, 11), and mesenchymal (12) and cardiomyocyte (10) stem cells. These findings raise questions on the role of pHi dynamics in morphogenesis and animal development, which remain largely unresolved. New genetically-encoded tools to measure pHi (51) and genetic and pharmacological approaches to selectively change pHi temporally and spatially will enable new studies necessary to resolve pHi-regulated developmental processes with promise for new approaches to correct impaired morphogenesis.

**Figure 2:**
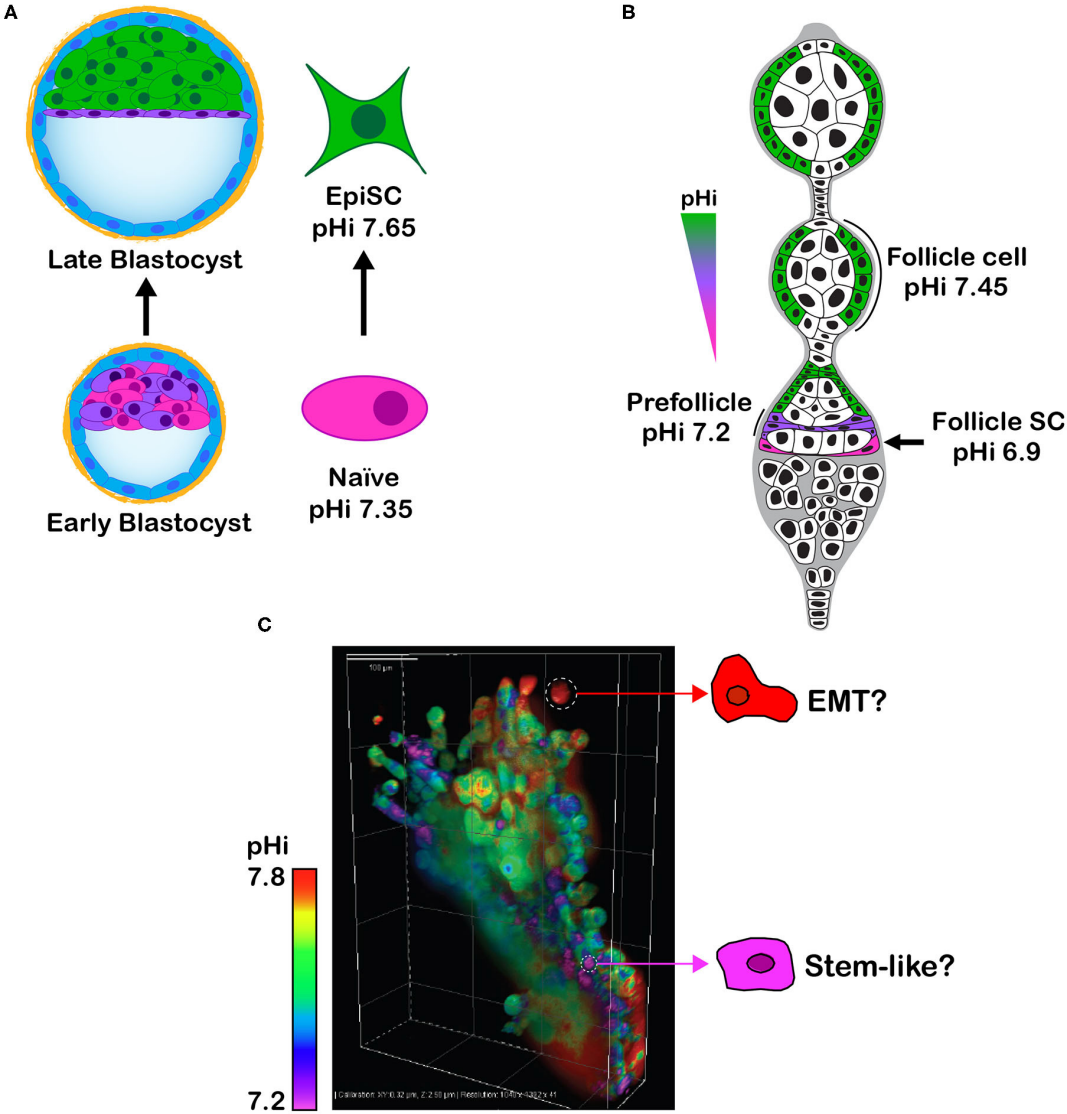
**(A)** Schematic showing that clonal self-renewing mESC (Naïve), derived from the inner cell mass of the early blastocyst, have a lower pHi than differentiated primed epiblast-like stem cells (EpiSC), which are analogous to cells in the late epiblast stage. **(B)** Schematic of Drosophila germarium showing an increase in pHi from self-renewing follicle stem cell (Follicle SC) to differentiated prefollicle and follicle cell. **(C)** Image of lung cancer H1299 cells expressing the pHi biosensor mCherry-pHluorin and grown in Matrigel as 3D spheroids shows intracellular pHi heterogeneity that might reflect phenotypic heterogeneity, such as cells with a higher pHi undergoing EMT and cells with a lower pHi being self-renewing tumor initiating stem-like cells.

Toward a goal of resolving the role of pHi dynamics in cell fate decisions, at the ISCaM meeting we discussed our findings on pHi-regulated embryonic and adult stem cell differentiation. As we previously described (11), with differentiation of naïve clonal mouse embryonic stem cells (mESC) to primed epiblast-like cells there is an NHE1-dependent transient increase in pHi of ~ 0.3 units (Figure 2A). Preventing this increase in pHi blocks differentiation, as indicated by sustained expression of the mESC markers Rex1, Stra8, and Nanog, and attenuated expression of the epiblast markers Brachyury, fibroblast growth factor 5, and Pax6. An increase in pHi is also necessary for differentiation of adult follicle stem cells in the Drosophila ovary to prefollicle cells and follicle cells (9, 11) (Figure 2B), the later necessary for germ cell maturation. Consistent with germ cells requiring enrichment from differentiated follicle cells, preventing the increase in pHi along the follicle stem cell lineage impairs ovary morphology and adult oogenesis and substantially decreases fertility (9). These findings were obtained by genetically silencing Drosophila *dnhe2*, an acid extruder, or overexpressing a newly identified Drosophila *ae2*, an ortholog of the mammalian acid loader AE2.

There are several important questions to resolve on the role of pHi dynamics in stem cell differentiation. First is whether pHi is a conserved regulator of stem cell differentiation in different tissues, perhaps using established and well-characterized models for intestinal epithelial (52) and skin epidermal (53) stem cell lineages. Second is how pHi dynamics regulates activity of pathways and functions of proteins with established roles in stem cell behaviors. One possibility is a role for pH sensing by β-catenin (as described above) in Wnt signaling, because high Wnt pathway activity (54) at low pHi may retain self-renewal of stem cells and inhibit differentiation. Third is whether pHi-regulated stem cell differentiation can inform regenerative medicine approaches to correct or restore impaired cell and tissue functions.

## Integrating pHi Dynamics in Cancer and Stem Cells

To consider how pHi dynamics in stem cells and cancer might be linked, we concluded our presentation by showing new data on pHi heterogeneity in spheroids of clonal human lung cancer cells (Figure 2C). Using H1299 cells expressing the previously described (41) genetically encoded and ratiometric pH biosensor mCherry-pHluorin, we observe distinct intercellular differences in pHi when grown in 3D (Figure 2C). Distinct pH heterogeneity (including intracellular and extracellular pH) is seen in cancer spheroids (55–58) and a mouse model of breast ductal carcinoma (59); however, whether this heterogeneity reflects differences in mutational signatures, cell identity, phenotypes, or epithelial or metabolic plasticity remains unresolved. For example, might cells with a lower pHi be stem-like tumor initiating cells? Could cells with a higher pHi have increased glycolysis to fuel rapid proliferation or be undergoing EMT for metastasis? The possibility that a lower pHi could enable tumor initiating cells raises caution on the idea of lowering pHi to limit cancer progression. Tumor heterogeneity, whether genetic, epigenetic, or phenotypic, is increasingly being recognized as a challenge for cancer therapies (60, 61), and improved understanding of the determinants and consequences of pHi heterogeneity could contribute to resolving these therapeutic challenges.

The field has taken a first important step in identifying a number of normal and pathological cell behaviors regulated by pHi dynamics. A second step in understanding how pHi regulates the signaling pathways mediating these behaviors is now emerging. A third step of improved mechanistic understanding is an important future direction to resolve design principles and functions of pH sensitive proteins mediating pHi-regulated cell behaviors. This third step is experimentally challenging and remains largely unexplored, but holds promise for identifying new therapeutic targets and informing the design of therapeutics for regenerative medicine and treating diseases with dysregulated pHi dynamics, including cancer.

## Author Contributions

All authors contributed to obtaining data included in the figures, including data on pHi and cancer (KW and DB), pHi and stem cell differentiation (YL and DB), and contributed to writing and editing the manuscript.

## Conflict of Interest

The authors declare that the research was conducted in the absence of any commercial or financial relationships that could be construed as a potential conflict of interest.

## Footnotes

^1^http://slc.bioparadigms.org/
